# DNA damage induced during mitosis undergoes DNA repair synthesis

**DOI:** 10.1371/journal.pone.0227849

**Published:** 2020-04-28

**Authors:** Veronica Gomez Godinez, Sami Kabbara, Adria Sherman, Tao Wu, Shirli Cohen, Xiangduo Kong, Jose Luis Maravillas-Montero, Zhixia Shi, Daryl Preece, Kyoko Yokomori, Michael W. Berns

**Affiliations:** 1 Institute of Engineering in Medicine, University of California-San Diego, San Diego, California, United States of America; 2 Department of Developmental and Cell Biology, University of California-Irvine, Irvine, California, United States of America; 3 Beckman Laser Institute, University of California-Irvine, Irvine, California, United States of America; 4 Department of Biomedical Engineering, University of California-Irvine, Irvine, California, United States of America; 5 Department of Biological Chemistry, University of California-Irvine, Irvine, California, United States of America; 6 Department of Physiology, University of California-Irvine, Irvine, California, United States of America; Tulane University Health Sciences Center, UNITED STATES

## Abstract

Understanding the mitotic DNA damage response (DDR) is critical to our comprehension of cancer, premature aging and developmental disorders which are marked by DNA repair deficiencies. In this study we use a micro-focused laser to induce DNA damage in selected mitotic chromosomes to study the subsequent repair response. Our findings demonstrate that (1) mitotic cells are capable of DNA repair as evidenced by DNA synthesis at damage sites, (2) Repair is attenuated when DNA-PKcs and ATM are simultaneously compromised, (3) Laser damage may permit the observation of previously undetected DDR proteins when damage is elicited by other methods in mitosis, and (4) Twenty five percent of mitotic DNA-damaged cells undergo a subsequent mitosis. Together these findings suggest that mitotic DDR is more complex than previously thought and may involve factors from multiple repair pathways that are better understood in interphase.

## Introduction

DNA damage occurs naturally through various endogenous and exogenous processes. Unrepaired DNA can compromise genetic integrity leading to developmental disorders, cell death or cancer. Organisms have evolved a variety of pathways to respond to the damage. The vast majority of studies on DNA damage responses have been done during interphase of the cell cycle. However, understanding the DNA damage response (DDR) during mitosis is also important since mutations accumulated during mitosis can lead to chromosomal aberrations, genomic instability of daughter cells, senescence and eventual cell death [1–4].

Studies examining the extent of DDR activation and repair in mitosis have primarily assessed the cellular response to double strand breaks (DSBs). DSBs may be repaired by homologous recombination (HR) and non-homologous end joining (NHEJ). HR preserves genetic fidelity as it relies on a homologous template to restore the damaged DNA. On the other hand, NHEJ leads to ligation of broken ends which can lead to loss of genetic information. Studies examining the DDR of DSBs in mitosis found truncated DDR that does not lead to the accumulation of ubiquitin ligases as well as 53BP1 and BRCA1 at mitotic damage sites [2, 5–10]. Subsequent studies revealed that mitosis-specific phosphorylation of 53BP1 by polo-like kinase 1 (PLK1) block 53BP1 binding to chromatin [11, 12]. Furthermore, RAD51 and filament formation were found to be inhibited by CDK1 in mitosis [13, 14]. Taken together, DSB repair, both NHEJ and HR, were thought to be inhibited in mitosis.

Further, DNA synthesis has been investigated in early mitosis with respect to DNA damage resulting from replication stress. However, this form of repair has been shown to be dependent on a process that is only activated in very late G2/early prophase [15–22]. Cells treated with Aphidicolin in S phase had Rad52 and MUS81-EME1 dependent DNA synthesis at chromosome fragile sites during very early prophase [16, 17]. The MUS81-EME1 complex and BLM helicase are required for the restart of DNA synthesis after replication stress [18–20]. Interestingly, cells synchronized to prometaphase did not undergo replication stress induced DNA repair synthesis [16, 17]. Additionally, MUS81 is associated with chromosome fragile sites in prophase but at a decreased rate in metaphase [18]. This form of DNA synthesis has been termed MiDAS (Mitotic DNA repair synthesis) and should not be confused with the DNA repair synthesis described in this paper. The mechanism of replication stress induced DNA synthesis likely differs from that observed in response to damage elicited by other means and from damage induced in other phases such as prometaphase, metaphase and anaphase. Thus, the ability of mitotic cells to undergo DNA repair synthesis in response to damage elicited during mitosis remains to be elucidated.

The majority of DDR studies have utilized ionizing radiation or radiomimetic drugs to induce DNA damage and study the subsequent mechanisms of DNA repair. These methods of DNA damage induction result in genome-wide alterations that may lead to different DDRs. However, the laser has demonstrated to be very useful for DNA damage response studies because of its ability to target a submicron region within a specified chromosome region [23–29]. Interestingly, a laser micro-irradiation study conducted over forty years ago, showed that when the nucleolar organizer was specifically damaged in mitotic cells, a few of the irradiated cells were able to undergo a subsequent mitosis. Karyotype analysis revealed intact chromosomes with a deficiency of the nucleolar organizer [26, 30]. In today’s context, these results suggest that the cells most likely repaired mitotic DNA damage through NHEJ.

Studies by our lab and others have shown the ability of a diffraction-limited focused near-infrared (NIR) 780nm laser micro-beam to induce DSBs marked by γH2AX, phosphorylated Histone H2AX on Ser 139, and KU in both interphase and mitosis [29, 31–37]. In addition to γH2AX and KU at laser-damaged sites, we have demonstrated that ubiquitylation was also occurring at damage sites in mitosis [28, 29]. Though our findings differ from those that utilized ionizing radiation [5, 10], the laser micro-irradiation approach permits the visualization of proteins such as KU, that do not form ionization radiation induced foci (IRIF) [38]. Therefore, it is not surprising to see accumulation of DDR proteins at laser damaged regions in mitosis that have been previously thought to be excluded from mitotic DNA damage.

In the current study we systematically characterize the nature of mitotic DNA damage induced by the NIR laser and perform quantitative analysis of DNA repair in mitosis and subsequent G1 phase in human and rat kangaroo (*Potorous tridactylus*) cells. Under our conditions, the NIR laser micro-irradiation of mitotic chromosomes induces complex damage consisting of both double strand breaks (DSBs) and single-strand breaks (SSBs) and ultraviolet (UV)-crosslinking damage (pyrimidine dimers) similar to what was recently described for damage to interphase cells [39]. We demonstrate that factors from various repair pathways whose function is better understood in interphase are capable of responding to mitotic DNA damage. Our results also indicate that DSBs generated on metaphase chromosomes lead to clustering of various proteins involved in NHEJ and HR. We show that DNA repair of mitotic DNA damage is ongoing and persists into G1.

## Materials and methods

### Reagents

See Supplemental S3 for a list of antibodies. Other reagents are listed under corresponding methods below.

### Cell lines

Five human cell lines were utilized in this study. U-2 OS cells, referred to as U2OS in this paper, are an osteosarcoma cell line ATCC HTB 96 that was used for the majority of studies unless otherwise mentioned. CFPAC-1 ATCC CRL 1918, a line derived from cystic fibrosis pancreatic adenocarcinoma was also utilized. The isogenic cell lines, M059K ATCC 2365 and M059J ATCC 2366 were utilized to compare the mitotic DNA response when DNA-PKcs is absent (M059J). M059K contains the wild type form of DNA-PKcs making this line a good control for the DNA-PKcs mutant. Both cell lines come from a glioblastoma in the same patient. Rat kangaroo cells (PtK2) from the Potorous tridactylus were utilized due to their large chromosomes and strong adherence to the substrate that facilitate mitotic studies. These cells were grown in Advanced DMEM/F12 with 1% Glutamax and 10% Fetal Bovine Serum. All Human cells were grown in Advanced DMEM supplemented with 1% Glutamax and 10% Fetal Bovine Serum. All cells were maintained in a humidified 5% CO_2_ incubator. Cells were plated onto glass bottom gridded dishes from MatTek to a confluency of 40% and used 1–2 days post subculture. Experiments were carried out in medium containing 40ng/mL nocodazole. For inhibitory experiments the following concentrations were utilized: ATM inhibitor (10μM KU55933), DNA PKcs inhibitor (3μM NU7441), and PARP inhibitor (100μM Nu1025).

### Laser induced DNA damage and microscope image acquisition

Mitotic chromosomes in live cells were irradiated using diffraction-limited (0.5–1 μm diameter) focal spots with a Coherent Mira 76 MHz 200 femtosecond micro-pulsed laser emitting at 780nm (Coherent Inc., Santa Clara CA). A series of beam expanders and mirrors coupled the beam into the right-side port of a Zeiss Axiovert 200M inverted microscope. An X-Y fast scanning mirror was positioned in the beam path prior to entry into the microscope port to facilitate moving the focused laser beam toward the desired target. The beam was focused through a 63x (1.4 NA) Zeiss Plan-Apochromat oil objective. The irradiance of the laser was controlled through the use of a Glan-Thompson polarizer mounted on a motorized rotational stage. A Uniblitz mechanical shutter controlled the exposure time of the laser. A single chromosome within the cell was targeted by the laser unless otherwise noted. Chromosomes were exposed to the laser for a total of 10ms within the focal spot. This exposure resulted in 7.6x10^5^ pulses of light to a spot measuring 0.68μm in diameter at the selected irradiance of 2.8–3.2x10^11^W/cm^2^.

Images were collected using a Hamamatsu CCD Orca [33, 40, 41]. The polarizer, scanning mirror and shutter were controlled by software developed with LabView [42]. To determine the irradiance at the focal point, the transmission of the objective was measured using a modified dual objective method described previously [28]. The objective used in these experiments had a transmission of 0.50 at the laser wavelength used. Based upon the measured irradiance of 2.8–3.2x10^11^W/cm^2^, the damage mechanism is likely of a multiphoton nature, either 2-photon or 3-photon, or a combination of both.

### Immunostaining

Cells were fixed with 4% paraformaldehyde in phosphate buffered saline for 20 minutes. Time to fixation after laser exposure varied according to the experiment. Cells were permeabilized overnight with blocking buffer containing 0.1% TritonX and 5% fetal bovine serum in phosphate buffered saline followed by staining with primary antibodies. Supplemental S3 shows a list of antibodies used. For most primary antibodies a 1:500 dilution was applied. Secondary antibodies against primaries were Alexa-488 goat anti-mouse (Invitrogen, Carlsbad, CA), and Cy3 goat anti-rabbit (Invitrogen, Carlsbad, CA) at dilutions of 1:2000.

### DNA synthesis detection

To test for repair/DNA synthesis, cells were incubated with 10**μ**M EdU (5-ethynyl-2’-deoxyuridine) 1-20min before laser exposure. A 10mM EdU stock was prepared according to protocol (Invitrogen catalogue #C10339). Cells that require prolonged mitosis were concurrently incubated with colcemid and EdU between 1–20 minutes prior to irradiation. Cells were fixed at time points ranging from 10–120 minutes after irradiation with 4% paraformaldehyde in PBS for 5-10minutes, and followed by blocking buffer containing 10% fetal bovine serum and 0.2% Saponin in PBS for 30minutes

### Terminal deoxynucleotidyl transferase (TdT) dUTP Nick-End Labeling (TUNEL) assay

DNA end-breaks were detected at sites damaged by the laser in mitotic chromosomes by TUNEL assay; dUTP labeling of exposed 3’-ends of DNA strands. The assay was followed according to the manufacturers protocol (Roche Applied Science).

### Image analysis

Tiff images were analyzed and subsequently edited to enhance the contrast and intensity using Image J software [43]. Mean pixel intensities(MPI) for laser DNA damaged regions were measured prior to contrast enhancement. The background was identified as the region outside of the DNA damage area and the mean pixel intensity of this area was subtracted from the fluorescence intensity of the lines or spots containing DNA damage in order to calculate the average pixel intensity at the damaged region. Positive signal for fluorescent markers was based on mean pixel values being higher than the level of background at undamaged chromosomes. Raw image datasets can be found through UC San Diego Library Digital Collections: https://doi.org/10.6075/J08W3BQK [44].

### UV induced DNA damage

U2OS cells were subjected to 254 nm light from a UVG-11 Compact UV Lamp by placing the lamp directly over 50mm cell dishes for 20 seconds. The power of the lamp was monitored with a PM100 ThorLabs Power Meter equipped with a S120UV sensor. The average power was measured before each experiment and determined to be 6mW. Prior to UV lamp exposure cells were switched into phenol red free Hanks buffered saline (Invitrogen). After UV exposure cells were either immediately fixed with 4% Paraformaldehyde or placed into a 37 C incubator prior to fixation.

### Pyrimidine dimer quantification

Mitotic U2OS were collected via mitotic shake off after synchronization with 9uM CDK1 inhibitor (Calbiochem; RO-3306). CDK1 inhibition results in arrest at G2; upon removal of inhibitor cells entered mitosis. Collected cells were exposed to 265nm UV light from a UV lamp UVG-11 (Science Company) for 20s in phenol red free medium. Cells were then separated into three aliquots and lysed at 30, 60 and 90 minutes after UV exposure. DNAzol Reagent (Life Technologies) was added to each sample for lysis and DNA isolation according to the manufacturer’s protocol. Isolated DNA samples were quantified using a Nanodrop 2000c (Thermo Scientific) and diluted to a working concentration of 2.0ug/ml in cold PBS. DNA samples were further diluted to plate on 96-well DNA High-Binding Plates. An OxiSelect UV-Induced DNA Damage ELISA Combo Kit was utilized to determine the concentration of pyrimidine dimers in each sample well (Cell Biolabs Inc). Plates were read with a Biotek Conquer ELX800 plate reader (Biotek Inc).

### Statistical analysis

Prism 7 for MAC OS was utilized for all statistical analysis. A T test was performed for comparisons to controls unless otherwise noted. Values were considered significant if P <0.05. Box plots show the 95% confidence intervals. Raw numbers and quantifications can be found in quantifications.xls under supplemental information. Images used for quantifications can be found at the following location: https://doi.org/10.6075/J08W3BQK [44].

## Results

### The laser induces complex DNA damage on mitotic chromosomes

**Fig 1:**
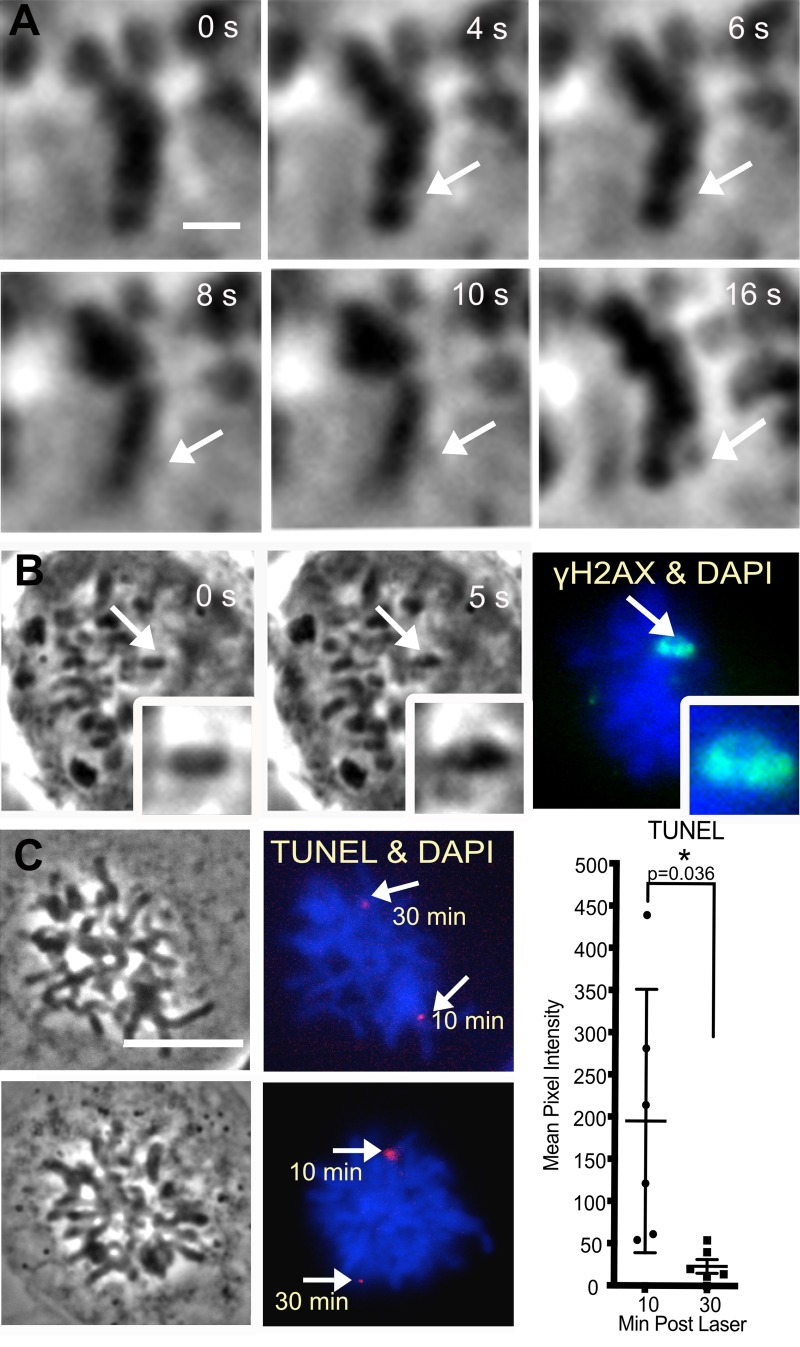
Characterization of laser induced DNA damage. (A) At the selected irradiance of 3.0x10^11^W/cm^2^ phase contrast changes are observed at a laser damage site on prometaphase chromosomes in nocodazole synchronized U2OS cells (4 and 6s). At 16s post laser (see arrows) phase dark material is apparent. Scale bar = 1μm. (B) γH2AX forms around the chromosome region damaged by the laser. A metaphase cell was fixed ~5s after the laser and immnunostained for γH2AX (arrows and inset). This cell was fixed prior to dark material accumulation. Paling is observed in the inset. (C) Positive TUNEL is seen at laser damaged regions (arrows) in prometaphase cells incubated with nocodazole. Two different chromosomes were damaged at different time points within the same cell. The first damage was induced 30 minutes prior to fixation and the second damage was induced 20 minutes after the first damage and fixed after 10 minutes. The TUNEL signal at the second damage site was brighter than the signal at the first damage. A graph of TUNEL signal on the right is the average of six cells per category, n = 6. The average and standard error mean of the late cut (fixed 10 minutes post laser) is 195 ± 61 and 23 ± 8 for the early cut (fixed 30 minutes post laser). p = 0.0363 Scale bar = 1цm.

Optimal laser parameters for the detection and consistent production of DNA damage in mitotic chromosomes were obtained by (a) varying the irradiance and comparing phase contrast image changes, and, (b) assaying for Nbs1 accumulation and γH2AX production. For this study we utilized irradiances of 2.8–3.2x10^11^W/cm^2^ unless otherwise noted. These irradiances allowed us to immediately determine that chromosomes were effectively damaged due to rapid phase contrast changes in the irradiated chromosome regions (Fig 1A arrows at 4 and 6s). Dark material, which may reflect phase separation is visible at 16s post laser exposure. In a different example, of a cell fixed ~5s after the laser, we see that γH2AX surrounds an area targeted by the laser (Fig 1B). In a previous study we showed that dark material is a result of the accumulation of DDR proteins and that γH2AX may surround the proteins and or overlap with them [34]. Additionally, at these irradiances γH2AX and Nbs1 were detected nearly 100% of the time (48 of 48 cells) and (16 of 17 cells) respectively.

The type of DNA damage induced by the laser on mitotic cells was assessed by immuno-staining for (1) DNA damage response proteins, (2) damaged bases, and (3) the TUNEL assay. Experiments were carried out in human U2OS cells unless otherwise mentioned. Positive TUNEL at laser induced DNA damage sites demonstrated the presence of END breaks, Fig 1C (magenta). TUNEL signal was higher in chromosomes fixed 10 minutes post laser when compared to those fixed 30 minutes post laser. These results indicate that factors may have bound broken ends.

Several DDR proteins were found at damaged chromosomes which provide information on the type of damage created by the laser. The consistent production of γH2AX demonstrates the lasers ability to induce DSBs. Pyrimidine dimers (CPD) as a result of the laser exposure were also detected and will be discussed further in this paper. XRCC1, which is involved in single strand break (SSB) repair, nucleotide excision repair (NER) and base excision repair (BER) localized to laser-damaged DNA (S1A Fig) [45]. However, we failed to detect significant base damage at laser-targeted regions at the irradiance range of 2.8–3.2 x10^11^ W/cm^2^ using an antibody specific for 8-Oxoguanine (8-oxoG) (Trevigen) (S1B Fig). Nevertheless, a key component of BER, APE-1 was detected at laser damage sites, S1C Fig.

### Mitotic cells undergo DNA repair synthesis

**Fig 2:**
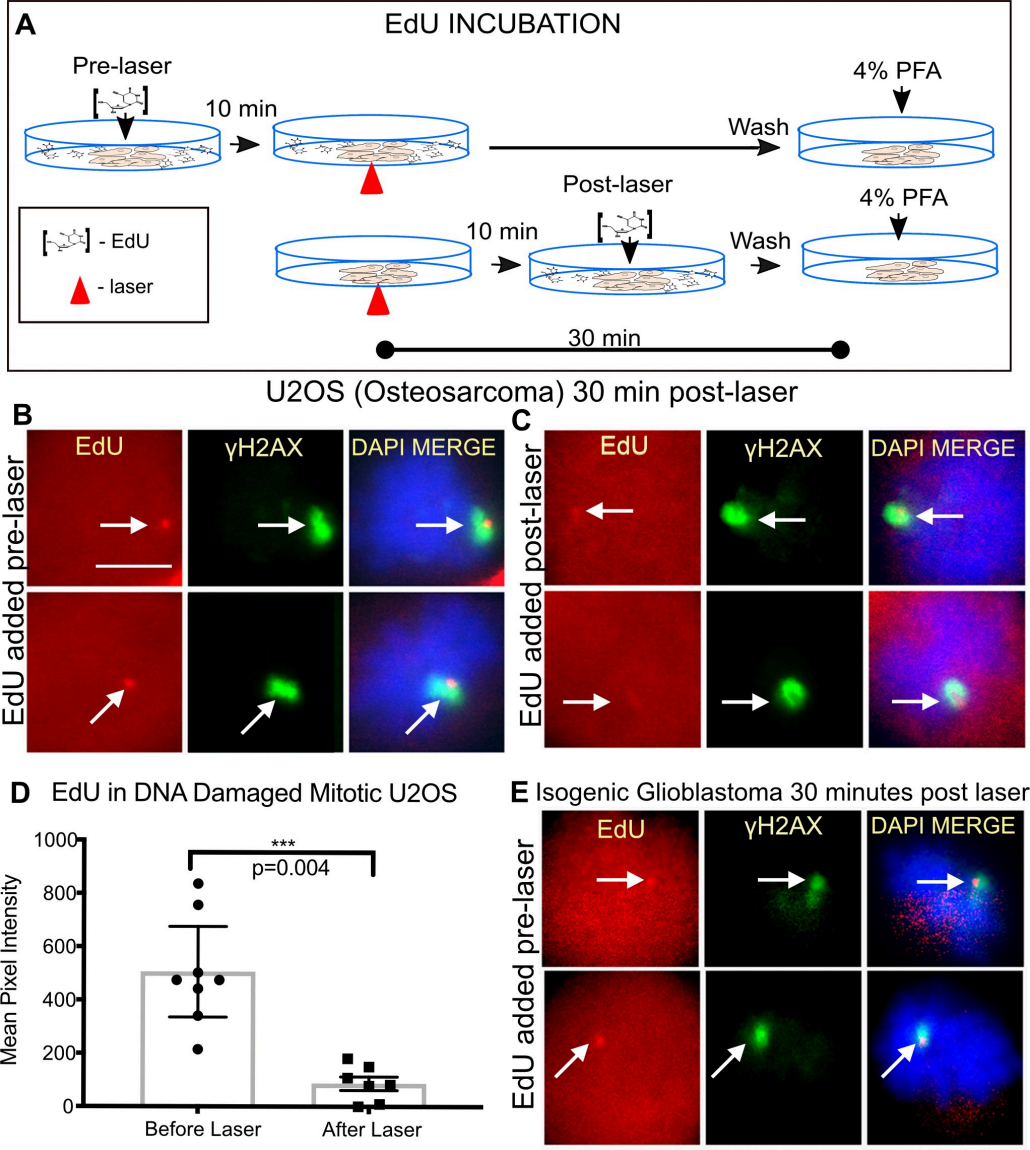
Mitotic cells undergo DNA synthesis repair. (A) Schematic of EdU incubation for experimental results shown in (B-E). In the first scenario, EdU was added to cells 10 minutes prior to the laser to allow penetration into cells before damage. In the second scenario. EdU was added to cells 10 minutes post laser to test whether repair is ongoing. All cells were fixed 30 minutes post laser. (B)Fluorescence images of nocodazole synchronized U2OS (Osteosarcoma) cells damaged under the conditions depicted in the schematic above were stained for EdU and γH2AX. γH2AX partially overlaps and surrounds EdU. (B and C) Two representative cells per condition are shown. Scale bar = 10цm (D) Quantifications of EdU intensity at the damage site in cells incubated pre or post laser. The average pixel intensity of EdU in cells pre-incubated with EdU was 505 ± 72 and 87 ± 25 for those incubated after the laser, n = 8 and 7 respectively. p = 0.0004 (E) Isogenic Glioblastoma cells M059K (top panel) and M059J (bottom panel) were stained for EdU incorporation and γH2AX.

Since DNA repair generally has been perceived to be inhibited in mitosis, we directly monitored the repair event in mitotic cells by the incorporation of the thymidine analogue 5-ethynyl-2’-deoxyuridine (EdU). For these experiments, cells were incubated either 10 minutes prior to laser or 10 minutes post laser. For both conditions cells were fixed 30 minutes post laser (Fig 2A). Cells damaged in metaphase were capable of incorporating the analogues, and this incorporation was surrounded by γH2AX known to spread to the neighboring chromatin [46, 47]. Two example cells per condition are shown in Fig 2B and 2C. Significant repair occurred during the first 10 minutes post-laser, Fig 2B. DNA repair synthesis is not restricted to the initial 10 minutes post laser as cells incubated with EdU post laser were still positive for EdU albeit weaker demonstrating ongoing DNA synthesis, Fig 2C and 2D. The incorporation of the EdU (i.e. repair) was observed in multiple cell lines during mitosis: U2OS (Fig 2B and 2C), and the Isogenic cell lines M059K & M059J (Fig 2E top panel and bottom panel, respectively).

### Mediators of DSB repair pathways

In an effort to identify what components of the DNA damage response may be active following laser damage to mitotic chromosomes, several damage sensors, adaptor proteins and transducers were tested for their ability to cluster to laser damage sites. Our evaluation begins with the DSB response. DSBs are amongst the most deleterious in that they can result in chromosomal translocations if left unrepaired [48].

**Fig 3:**
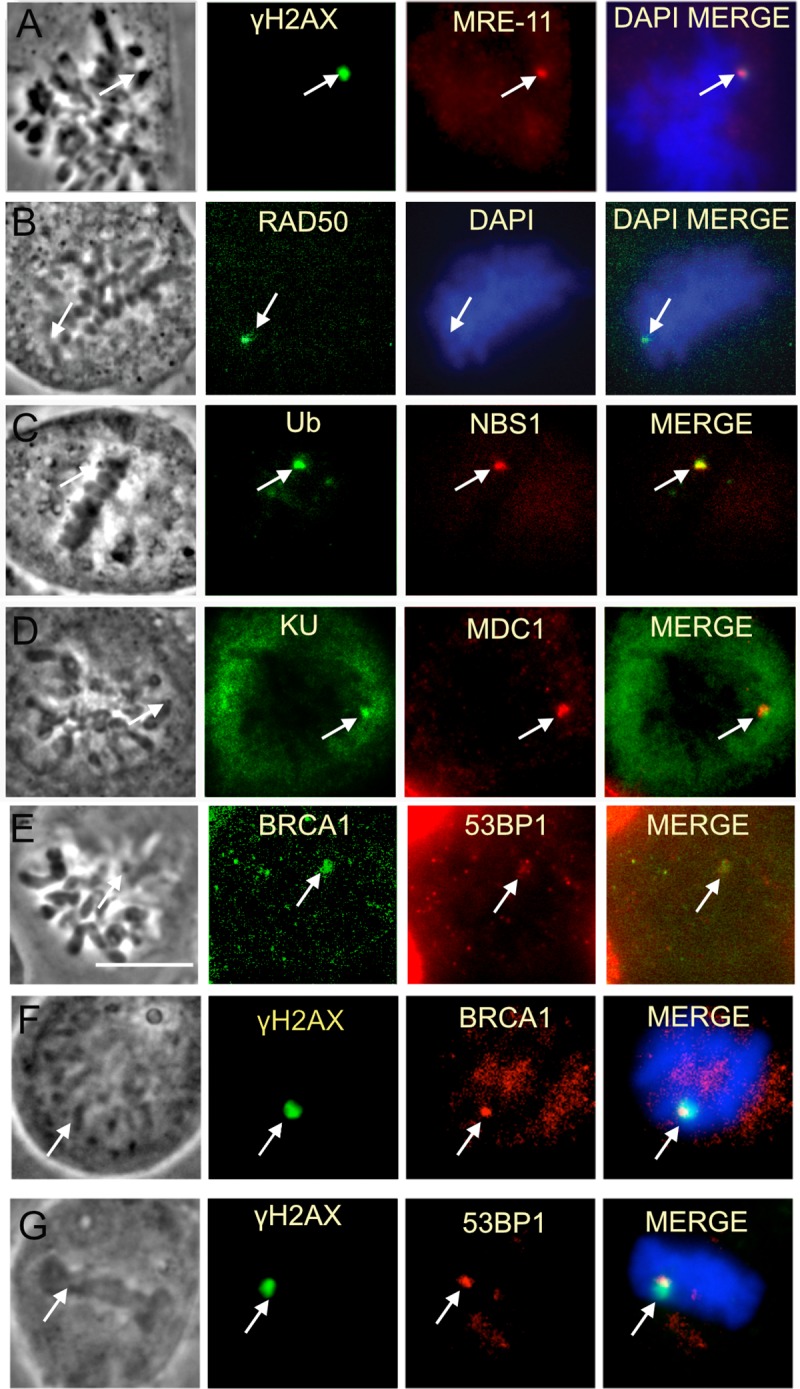
Mitotic laser induced DNA damage leads to the recruitment of various DDR proteins including some not previously observed on mitotic DNA damage. Cells in this figure were fixed within 30 minutes of laser damage. (A-C) The MRN complex (MRE-11, Rad50, Nbs1) forms at laser damaged chromosomes. Nocodazole synchronized U2OS cells are shown. MRE-11, Rad50, NBS1. (C-E) Ub, KU, MDC1, 53BP1 and BRCA1 were also observed at chromosomes damaged by the laser. Scale bar = 10μ m. (E and F) BRCA1 immunostaining using two different antibodies. (E) BRCA1 ab16780 antibody (Abcam). (F) BRCA1 OP107 antibody (Calbiochem). (F-G) show the localization of BRCA1 and 53BP1 with respect to yH2AX.

During interphase the MRE11-RAD50-NBS1 (MRN) complex is one of the first factors to recognize DSBs [49, 50]. At the selected irradiance all three components of the MRN complex are detected at laser-induced damage sites in mitotic cells (Fig 3A–3C). Consistent with the presence of γH2AX, MDC1, which binds to γH2AX [51], was also recruited (Fig 3D). Although, BRCA1, 53BP1 and Ubiquitin (Ub) responses have been observed to be attenuated in mitosis, [52, 53] we previously showed an Ub signal at NIR laser-induced damage sites in PtK1 cells [28]. In the present study, Ub accumulation was also observed in mitotic U2OS cells (Fig 3C). Ub response at damage sites is critical for localization of BRCA1 and 53BP1 at DSBs in interphase nuclei [54, 55]. Correlating with the presence of the Ub signal at mitotic damage sites, we observed the accumulation of BRCA1 and 53BP1 at mitotic damage sites (Fig 3E–3G). Our results indicate that Ub, BRCA1 and 53BP1 can be recruited to highly clustered laser-induced damage sites on mitotic chromosomes.

### Complete assembly of non-homologous end joining factors in response to mitotic DNA damage

**Fig 4:**
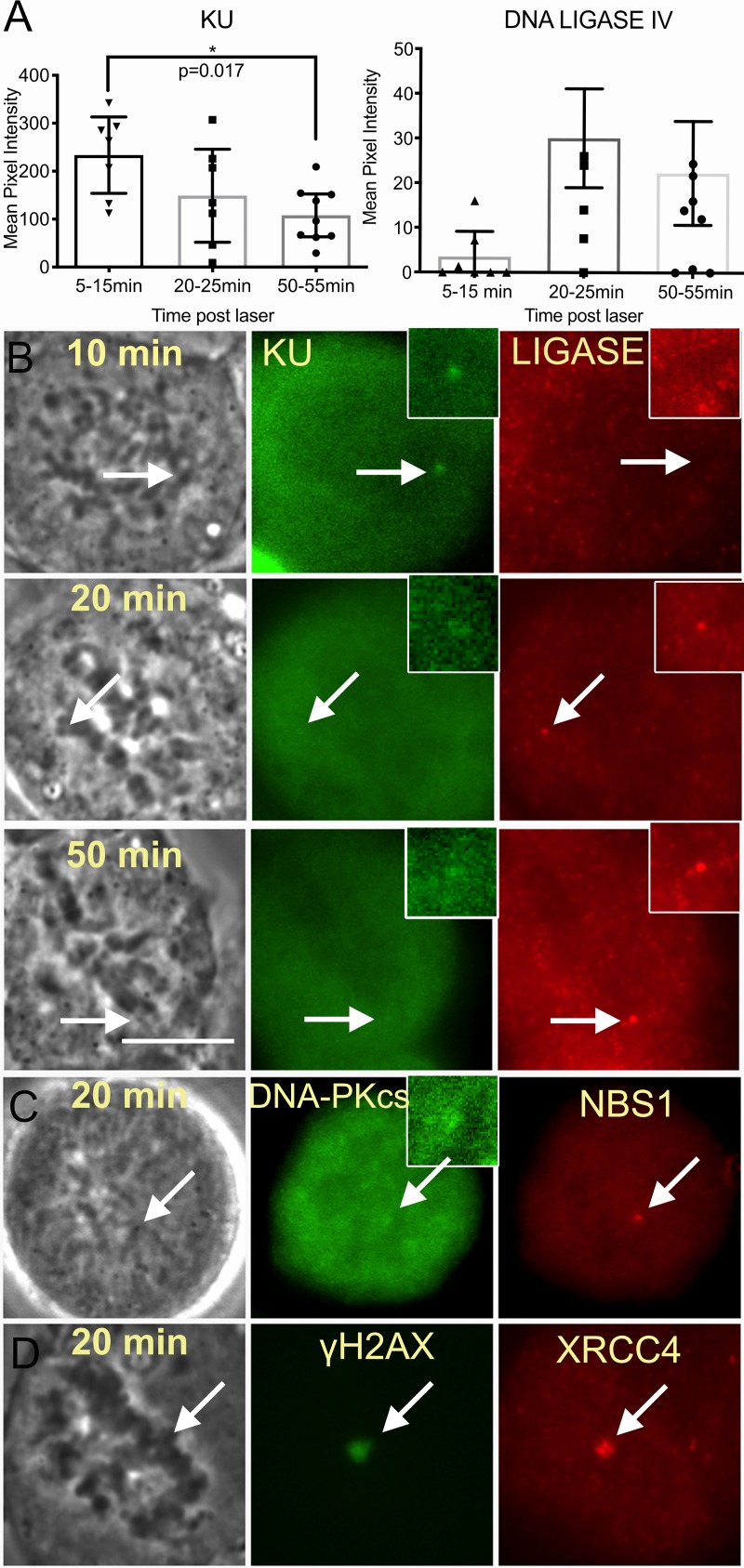
NHEJ factors cluster at mitotic DNA damage. (A) KU is localized to damaged chromosomes in cells fixed 5–15 minutes post laser. A box plot shows the signal intensity of KU and LIGASE IV immunostained U2OS cells. Cells were maintained in nocodazole throughout experiments and fixed at: 5-15min, 20-25min and 50-55min. Mean values and standard error mean for KU: 236 ± 31 N = 7, 149 ± 40 N = 7 and 108 ± 19 N = 9 respectively. Mean values and standard error mean of Ligase under the same time intervals are: 3.5 ± 2.3 N = 7, 30 ± 11 N = 7, 22 ± 12 N = 9. Confidence intervals are shown for both graphs. (B) Images of KU and LIGASE IV stained U2OS fixed at 10, 20, and 50 minutes post laser. An arrow depicts the area targeted by the laser and a region that is magnified as an inset. Scale bar = 10 μm. (C) DNA-Pkcs clusters to damaged U2OS chromosomes. An inset depicts a magnified view of immunostained DNA-PKcs at the cut site. (D) Immuno-labeled XRCC4 and γH2AX are also seen at laser damaged region.

NHEJ initiation has been observed in mitotic DNA lesions using a NIR laser in two separate studies [28, 38]. In our studies KU accumulated during the first 5–15 min post laser (Fig 4A). In contrast, the recruitment of DNA ligase IV that mediates end joining at a later step of NHEJ was not apparent until 20 minutes post irradiation (Fig 4A and 4B). DNA PKcs and XRCC4, the binding partner of ligase IV, was also detected at laser damage (Fig 4C and 4D). Therefore, complete assembly of NHEJ factors may occur in mitosis.

**Fig 5:**
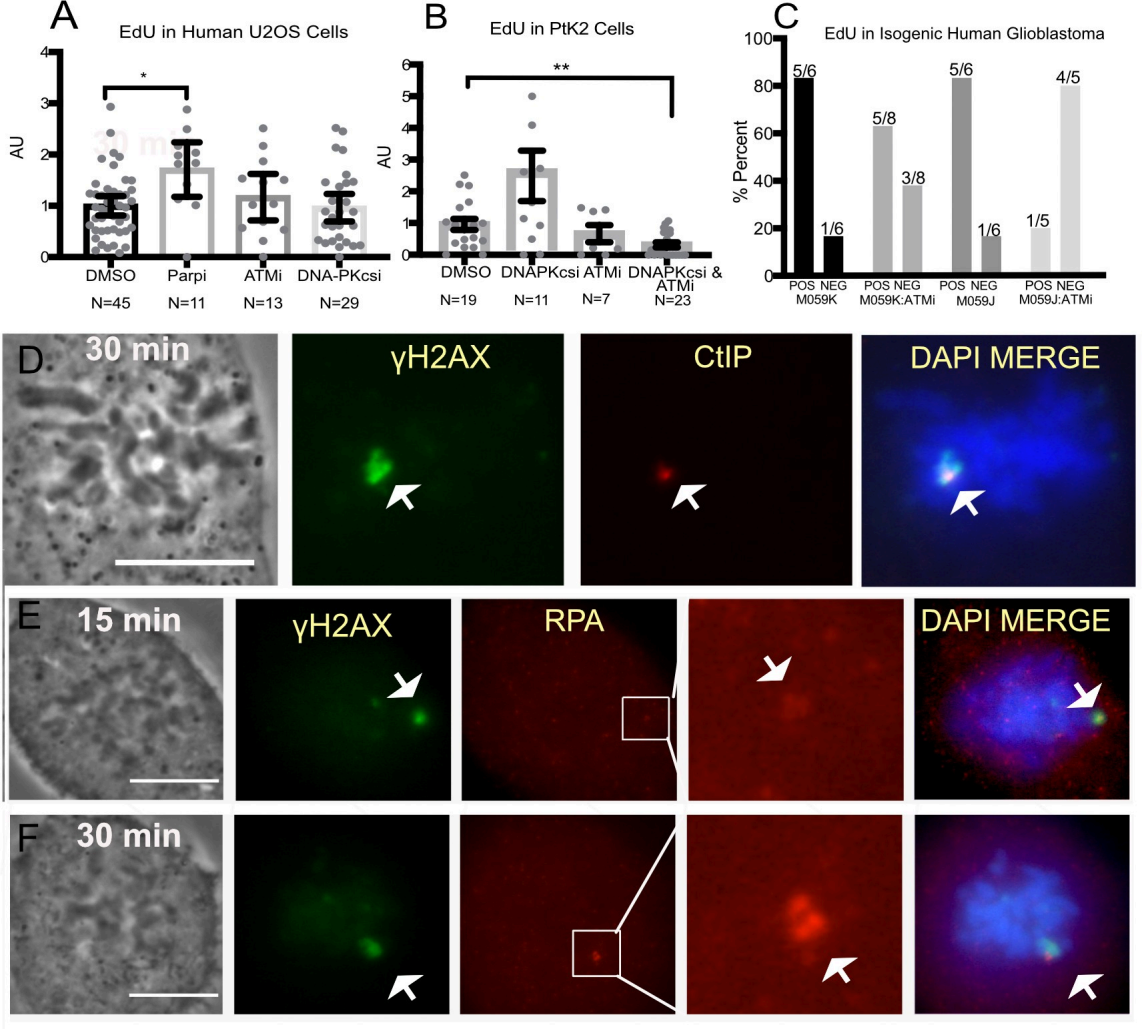
Mitotic DNA synthesis on DNA damaged chromosomes of cells with compromised ATM, DNA PKcs or PARP activity. (A) Nocodazole synchronized U2OS cells treated with inhibitors for PARP (100μM NU1025), ATM (10μM Ku55933) and DNA PKcs (3μM NU7741) were assessed for DNA synthesis. Each experiment was normalized to the mean of the corresponding DMSO control. (B) Synchronized PtK2 cells treated with DMSO, ATM inhibitor, and combined DNA-PKcs and ATM. (C) Nocodazole synchronized human isogenic cell lines M059K and M0959J (DNA-PKcs deficient) were treated with ATM inhibitor. The percent of positive cells is plotted. Above each bar is the number of cells per category. Values used for this graph are found in S4 Fig. EdU was fluorescently labeled by the Click-it assay to test for repair synthesis (D) CtIP and (E) RPA were found on laser-damaged chromosome regions of immunostained U2OS cells fixed 15 (top panel) and 30 (bottom panel) minutes post laser.

Classic NHEJ is dependent on DNA-PKcs. We investigated the contribution of NHEJ on DNA synthesis repair by utilizing DNA-PKcs deficient (M059J) and isogenic DNA-PKcs-positive (MO59K) cell lines. Immunostaining of DNA-Pkcs in the isogenic lines confirmed its presence in M059K and absence in M059J (S2A Fig). Nevertheless, DNA repair synthesis was observed in both cell lines (Figs 2E and 5B). Similarly, U2OS cells treated with 3 μM DNA-PKcs inhibitor NU 7441 showed no significant difference when compared to control cells (Fig 5A DMSO vs DNA-PKcs inhibitor).

Alternative-NHEJ (alt-NHEJ) may also repair DSBs when DNA-PKcs is compromised [56]. Poly (ADP-ribose) polymerase 1 (PARP1) has been shown to play a pivotal role in alt-NHEJ [57, 58]. Therefore, we inhibited PARP in U2OS cells with 100μM NU 1025 and tested for effective inhibition by immunostaining for poly(ADP-ribose) (S2B Fig). S2D Fig depicts an untreated U2OS mitotic cell positive with PAR staining at the damage site. Interestingly EdU results from cells synchronized with nocodazole suggest that PARP inhibition may lead to greater DNA synthesis (Fig 5A, DMSO vs PARPi). Since PARP inhibition is known to stimulate c-NHEJ, the effect of PARPi on EdU incorporation was examined in DNA-PKcs deficient cells [39, 59, 60]. PARP inhibition did not abolish DNA synthesis at mitotic DNA damage sites (S2C Fig). These results reveal that PARP signaling plays a role in suppression of DNA repair during mitosis.

### Homologous recombination is activated in mitosis and may initiate RAD51 filament formation in the absence of functional DNA-PKcs or CDK1

Homologous recombination may preserve genomic integrity during the repair of DSBs. This process relies on resection of the damaged DNA ends and a homologous template to synthesize new DNA and preserve genomic integrity. ATM kinase is key to the activation of this repair pathway [61, 62]. Simultaneous ATM and DNA PKcs inhibition attenuated DNA synthesis at mitotic damage sites in PtK2 cells (Fig 5B). Similarly, MO59J cells treated with ATM inhibitor had almost complete abolishment of EdU (Fig 5C). Since ATM was shown to stimulate HR repair in interphase [61, 62], these results raise the possibility that when both c-and alt-NHEJ pathways are inhibited, HR factors may contribute to DNA repair during mitosis.

A key first step towards HR repair involves DNA end resection through CtIP followed by RPA binding to the resected DNA ends [63]. Consistent with previous studies that used meiotic Xenopus extract to monitor DSB repair, we observed CtIP and RPA at mitotic laser damage sites suggesting ongoing end resection (Fig 5D and 5E) [64].

**Fig 6:**
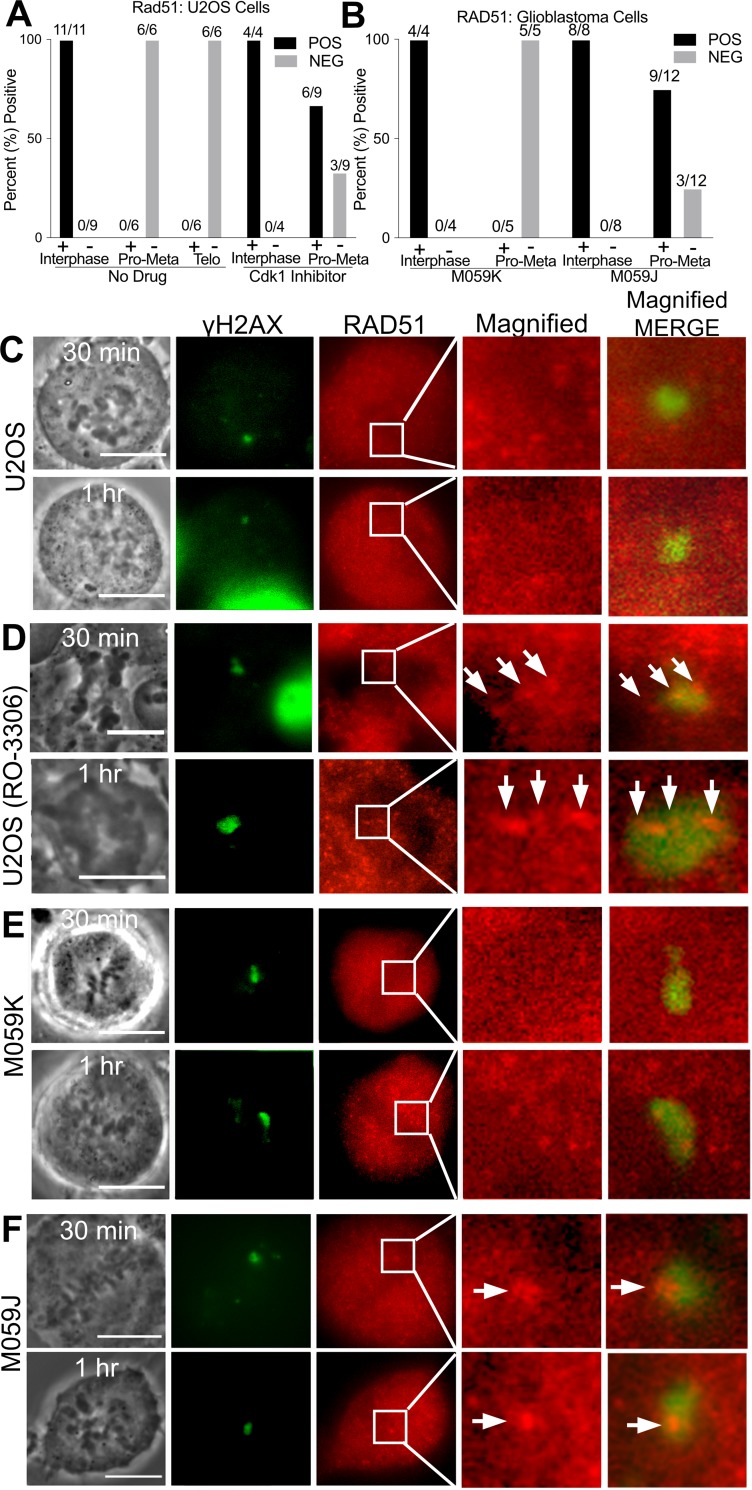
DNA damaged chromosome regions are devoid of RAD51. (A) Percent of U2OS cells fixed 25–90 minutes post laser that are positive for RAD51 according to mitotic phase based on immunofluorescence staining data. CDK1 inhibition causes some cells to present mean pixel values above background, MPI = 53 ± 16 in six out of 9 cells. Corresponding box plot can be supplemental files. (B) DNA Pkcs deficient cells, (M059J) also show a greater likelihood of RAD51 above background levels when compared to U2OS. Nine out of twelve had positive RAD51, MPI = 121 ± 67. Corresponding box plot can be found in supplemental files. C) U2OS mitotic cells stained for γh2AX and RAD51. (D) U2OS treated with 10 μM CDK1 inhibitor underwent premature cytokinesis and chromosome de-condensation. Nevertheless, RAD51 co-localized with γh2AX and appears dotted along a track in an enlarged image of a boxed region shown on the RAD51 column. (E) The isogenic glioblastoma lines M059K and (F) M059J were DNA damaged with the laser and stained for γh2AX and RAD51. (F) M059J cells (deficient in DNA-PKcs) show RAD51 at the damage spot. Scale bar = 10μm.

Downstream of end resection and RPA binding is RAD51 filament formation for homologous strand invasion. Previously, RAD51 was reported to not accumulate to damaged meiotic chromatin from *X*. *laevis* egg extract unless CDK1 was inhibited [64]. Similarly, we failed to observe RAD51 filament formation in U2OS mitotic cells synchronized with nocodazole (Fig 6A and 6C). In contrast, RAD51 accumulation was observed at the laser damage sites in interphase cells fixed at 25 minutes post laser damage. Similar results were obtained with a different human cell line, CFPAC-1, indicating that this is not a cell type-specific phenomenon (S2E and S2F Fig).

CDK1 was shown to play a role in HR inhibition in mitosis [9, 11, 64, 65]. Cells treated with 10μM CDK1 inhibitor R0-3306 underwent premature cytokinesis and or chromosome de-condensation within 5 to 10 minutes of inhibitor addition (Fig 6D). A proportion of CDK1 inhibited mitotic U2OS cells demonstrated slightly higher fluorescence pixel values above background (53 ± 16) than control cells whose values were negative (Fig 6A and 6D). RAD51 appears filamentous at damage sites showing positive RAD51 staining (Fig 6D, arrows on magnified view). However, the levels of fluorescence intensity were not in the same positive range of RAD51 (1305 ± 222 mean pixel intensity) seen in interphase cells. Thus, it would appear that other pathways independent of CDK1 activity may regulate RAD51 suppression.

Interestingly, in the absence of DNA-PKcs, most cells (9 of 12) showed some RAD51 at damage sites (MPI = 121 ± 67)). These results suggest that mitotic DNA-PKcs also regulates RAD51 accumulation at clustered damage sites (Fig 6B and 6F). Interestingly the staining pattern of RAD51 differs from that observed in CDK1 inhibited U2OS cells (compare magnified view i.e. 10x of Fig 6D and 6F). M059K cells did not show RAD51 that localized to the damage area (Fig 6E).

In contrast to NHEJ, we observed the accumulation of factors involved in only the early part of the HR pathway at DNA damage sites on mitotic chromosomes. CtIP and RPA both increase over time suggesting the formation of more strand breaks or end resection. In contrast, both Rad51 and cohesin recruitment was blocked (S2 Fig). We found that RAD51 binding is inhibited not only by CDK1 [13], but also by DNA-PKcs in mitosis (Fig 6). Lack of cohesin may be due to the fact that it is destabilized during mitosis and/or it only promotes HR between sister chromatids but not other types of HR [66, 67].

### Mitotic DNA repair synthesis is not a laser specific phenomenon

**Fig 7:**
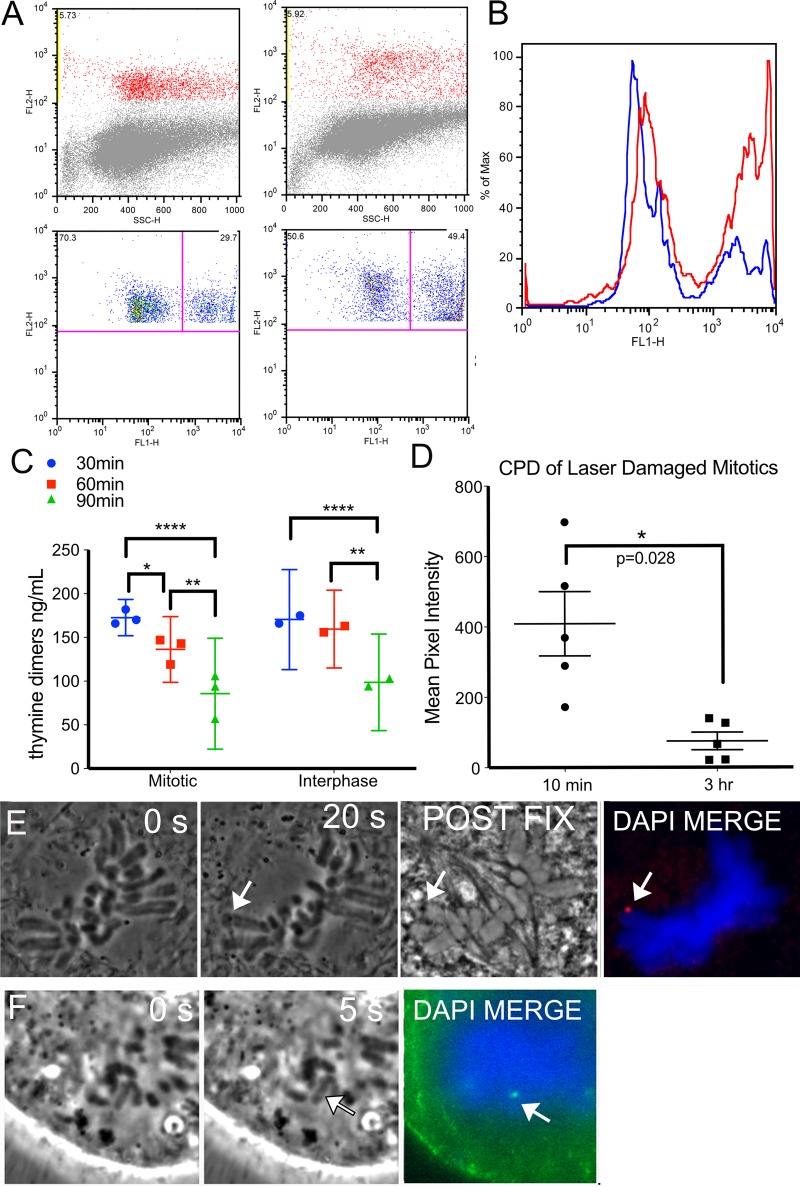
UV induced DNA damage repair in mitosis. (A) FACS of U2OS cells stained for mitotic marker, phospho-H3S10 (y-axis) plotted against side scatter (x-axis) on the top two panels. Cells that stained positive for phospho-H3S10 were plotted against EdU (x-axis) in the bottom panels. The right quadrant of each plot shows mitotic cells that stained positive for EdU. A greater proportion of cells are positive for EdU following UV exposure. Compare 30% without UV to 50% with UV. (B) A histogram of both populations, of cells, damaged/UV exposed in red and undamaged in blue to show the way the populations shift towards greater EdU signal after UV exposure. (C) ELISA of a population of non-laser UV exposed synchronized mitotic and interphase cells collected at 30, 60, and 90 minutes post exposure. N = 3 replicates for mitotic populations and N = 2 for interphase cells. (D) Quantification of CPD intensity in U2OS mitotic cells compared at 10 minutes and 3hours post laser, N = 5 per category. (E) A mitotic PtK2 whose chromosome was damaged by the laser(arrow). A post fixation phase image of the cell shows a dark spot at the laser cut site. Cyclo-butane pyrimidine dimers (red) are seen at the exposure site. (F) A U2OS chromosome positive for cyclo-butane pyrimidine dimers (green) at the damaged site.

Our EdU labeling results strongly indicate that there is repair of laser-induced damage in mitosis. To confirm that mitotic DNA synthesis repair can occur in cells damaged by other means we exposed cells to UV light from a lamp and then isolated by FACS using an antibody specific for phosphorylated histone H3 Serine 10 (phospho-H3S10) (Fig 7A red). Phospho-H3S10 is greatest during mitosis and therefore the mitotic population can be easily separated from the interphase population. Mitotic cells were plotted against EdU fluorescence intensity (Fig 7A, bottom panels). The scatter plots reveal that 50 percent of cells stained positive for EdU in response to UV when compared to 30 percent of control cells. A histogram of the same results in Fig 7B depict the increase in proportion of cells staining positive for EdU has increased (red histogram compared to blue). The results indicate that UV damage repair can occur in mitotic cells and mitotic repair is not a laser damage specific phenomenon. Amongst our findings are results showing that mitotic cells are capable of removing pyrimidine dimers (CPD) as confirmed through ELISA of UV damaged cells, Fig 7C. Similarly, cells damaged by the laser demonstrated a decrease of pyrimidine dimers, Fig 7D. PtK2 and U2OS cells stained for CPD are shown in Fig 7E and 7F, respectively.

### Mitotic DNA damage is carried into interphase

**Fig 8:**
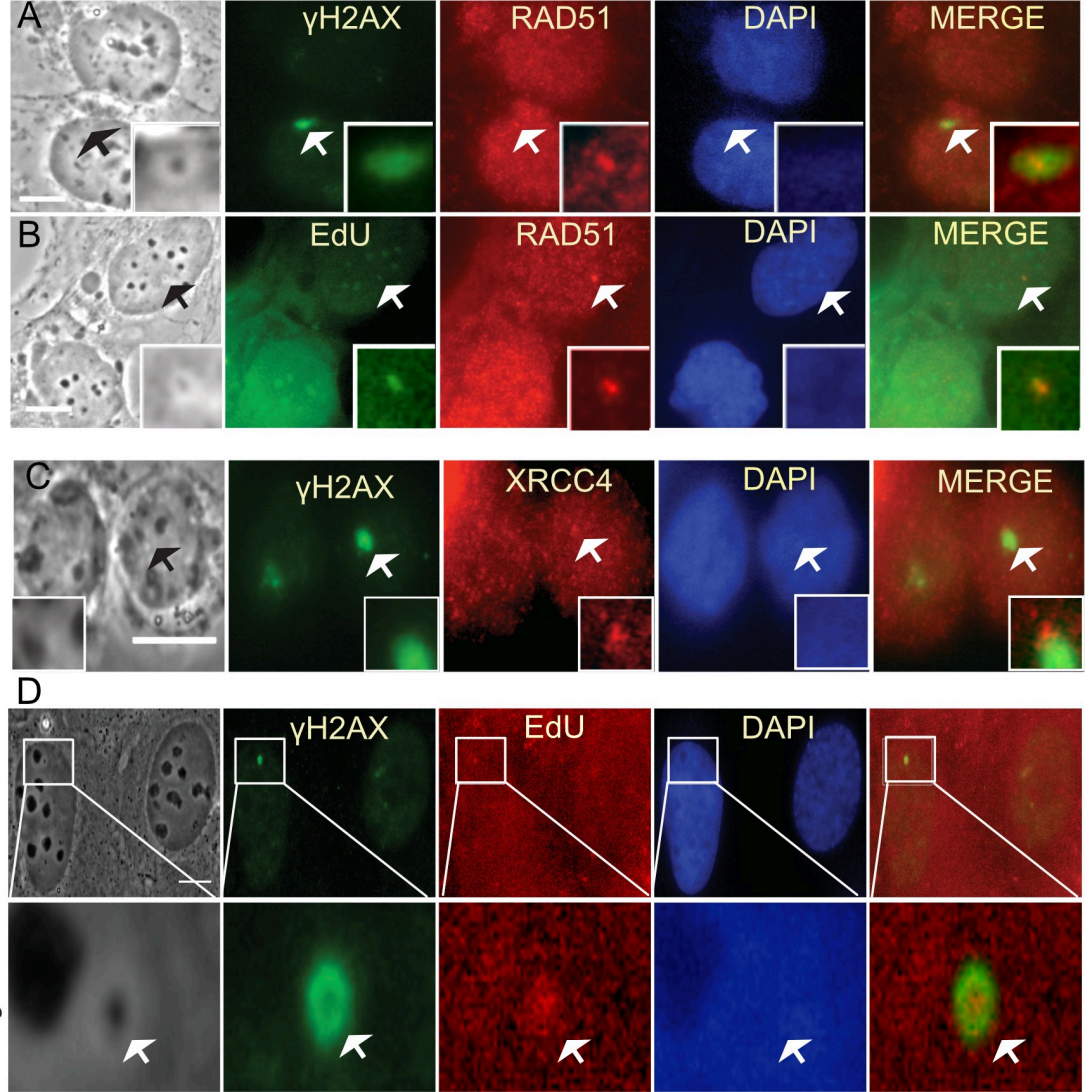
Mitotic DNA damage carried into G1 suggests ongoing repair. Cells damaged in mitosis where fixed 2 hours post division and immunostained for downstream HR and NHEJ factors as well as for EdU. (A) RAD51 and γH2AX localize to the same area of a G1 cell. (B) EdU and RAD51 co-localize with each other and a phase dark spot of a G1 cell. (C) XRCC4 and γH2AX slightly overlap in a G1 cell. Insets depict a magnified view of damage spots pointed out in arrows. (D) Forty hours post mitosis a cell has a phase dark spot that is surrounded by γH2AX and that co-localizes with EdU. Scale bar = 10μm.

The ability of clustered mitotic DNA damage to accumulate RAD51 post mitosis was investigated. RAD51 was not detectable in our studies unless CDK1 and or DNA-PKcs activity was compromised. Therefore, cells were examined for the ability of HR factors to recruit to laser damage created in mitosis once the cells had entered G1. We found that in the following G1 phase, RAD51 does accumulate to the DNA damage produced in the preceding mitosis (Fig 8A and 8B). EdU colocalized with RAD51 indicating the possibility that HR may be responsible for some of the incorporation and that repair is ongoing (Fig 8B and 8D). A cell damaged in metaphase and fixed 40 hours post mitosis is still undergoing DNA repair synthesis(Fig 8D).

Additionally, we assessed whether NHEJ factors are still present in G1 from damage created in mitosis. Immuno-staining for XRCC4 showed that, in fact, XRCC4 is still present at G1 (Fig 8C). Thus, it seems that the cell may be trying to repair clustered laser damage utilizing factors from NHEJ and HR. Previously we reported that BRCA1 and 53BP1 were also observed post mitosis [28]. This result further supports repair is ongoing.

We investigated the ability of cells damaged in metaphase, anaphase and G1 to complete mitosis and enter a subsequent mitosis. For this, undamaged U2OS cells followed under our culture conditions showed an average division time of 37 ± 5 hours post cytokinesis. Values were calculated by taking the time of cytokinesis and following a cell until its entry into the subsequent mitosis (S3 A controls, N = 11 cells). As a result, damaged cells were followed for a minimum of 40 hrs.

**Fig 9:**
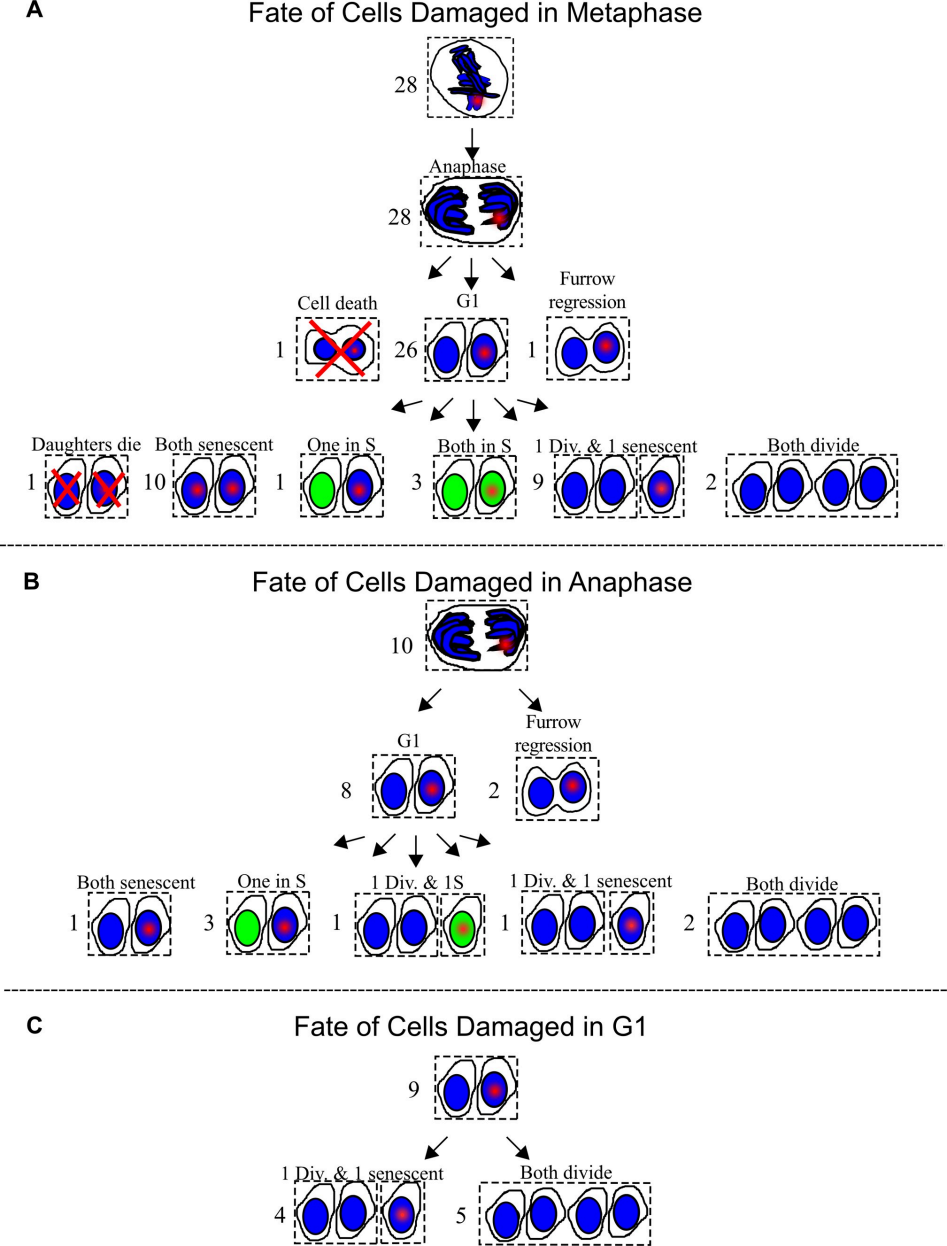
Fate of cells damaged in mitosis and G1. (A) Twenty-eight metaphase cells were DNA damaged by the laser. A red point depicts laser damage. Twenty six out of the twenty-eight divided. One underwent furrow regression. Another underwent cleavage formation followed by cell death. The fates of daughter sets are shown below. A green nucleus marks S-phase. Divides is abbreviated as div. Six different outcomes are summarized: 1) both daughters die, 2) both daughters are senescent, 3) one daughter is in S and another is senescent, 4) both daughters are senescent, 5) one daughter divided and another is senescent, and 6) both daughters divide. (B) Ten cells were DNA damaged during Anaphase. Eight cells progressed into G1. Of the two that did not progress into G1, one underwent furrow regression, and another appears to have fused at a later point. Five outcomes for the daughters are summarized: 1) one set had both daughters in senescence, 2) three sets had one in S and the other in senescence, 3) one had one daughter divide and another in S phase, 4) another had one daughter divide and the other was senescent and 5) two sets had both daughters divide. (C) Nine G1 sisters were identified. One sister from each set was damaged. The cell containing the damaged nucleus has a red point. All of the undamaged sisters divided. Five of the damaged sisters divided.

**Fig 10:**
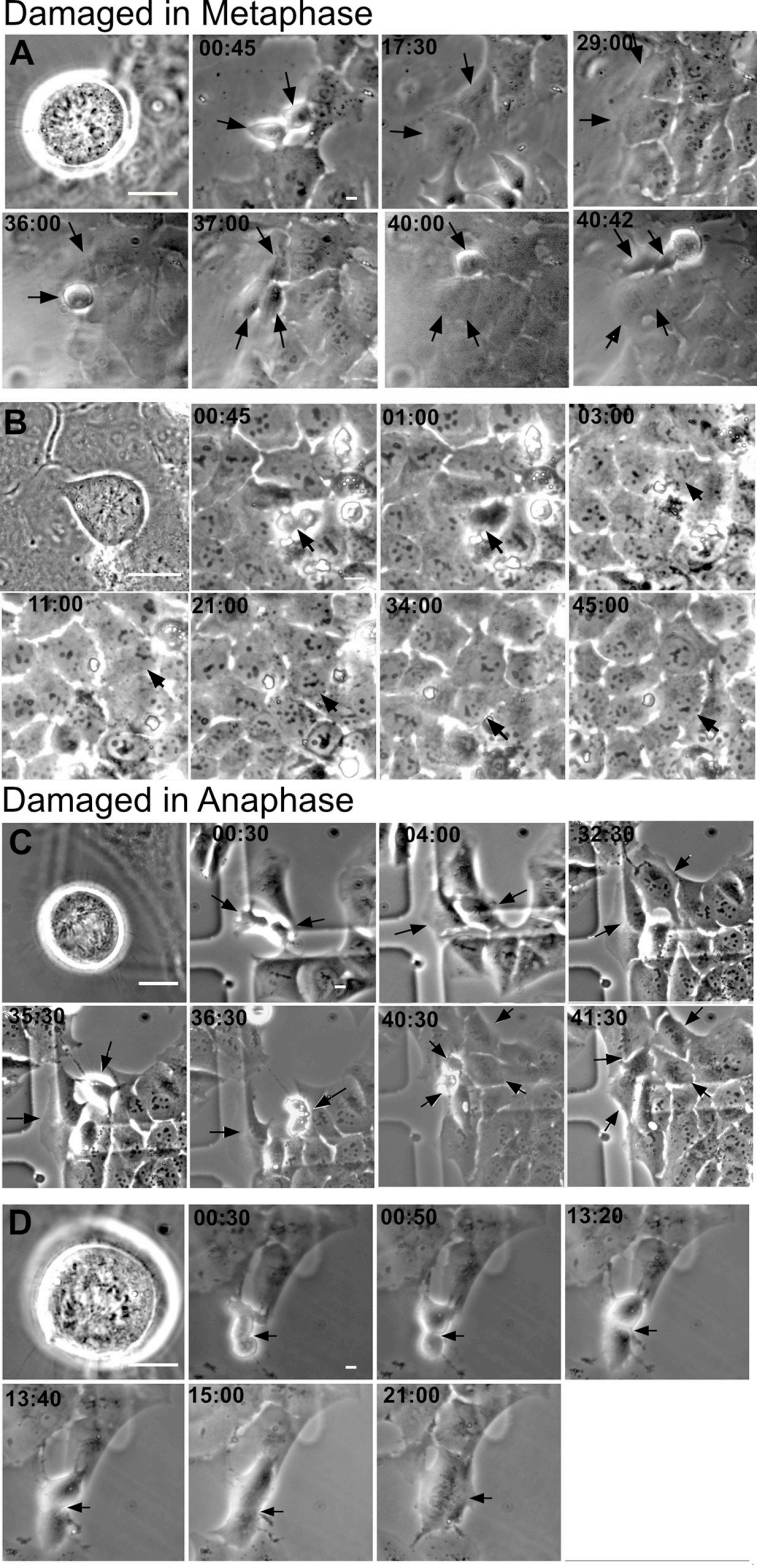
Time-lapse of cells damaged in mitosis. (A) Montage of a cell DNA damaged in metaphase whose daughters underwent mitosis at 36 and 40 hrs post division. Laser damage was created through a 63x objective. Therefore, cells appear larger on the first image. Subsequent images were taken with a 20x objective to broaden the field of view. (B) A metaphase DNA damaged cell whose furrow regressed at 1hr. (C) Montage of a cell damaged in Anaphase whose daughters divide at 36:30 and 40:30 hours. (D) An anaphase cell that appears to have divided, see 00:50 and 13:20. However, at 13:40 and 15:00 the cell begins to show furrow regression. Scale bar = 10μm.

The majority of mitotic cells damaged with the laser entered G1, 26 of 28 of cells damaged in metaphase and 8 of 10 cells damaged in anaphase (Fig 9A and 9B). One cell damaged in metaphase died. The daughters of cells that divided were followed and entry into mitosis i.e. completion of a cell cycle was assessed within an observation window up to 40–47 hours post DNA damage. A proportion (21%) of daughter cells with damage inflicted in pro-metaphase underwent a subsequent mitosis, compared to 37% for daughter cells carrying damage elicited in anaphase. Examples of time-lapse analysis of cells damaged in metaphase and anaphase are shown in which both daughter cells underwent subsequent mitosis (Fig 10A and 10C). Cells that did not divide and reverted are shown in Fig 10B and 10D. Daughter cells carrying the damaged chromatin took longer to divide than their counterparts without damaged chromatin (S3B Fig).

Damaged G1 cells were also followed to compare their ability to repair with that of mitotic cells. These cells were identified by following anaphase cells until completion of division and formation of two daughter cells. Of both daughter cells only one sister was damaged with the laser. However, both sisters were followed. Prior to fixation, all cells were incubated with EdU to check for S-phase status of cells that had not divided within the observation window. Fig 9 contains a summary of cell fates.

As expected, all undamaged G1 sisters entered mitosis within the observation window. Out of the nine damaged cells, five divided i.e. 55% divided. Our results suggest that damage in metaphase is more deleterious than damage induced in anaphase or G1 (Fig 9C). Notwithstanding, these results demonstrate that a percentage (25%) of cells laser damaged in mitosis (metaphase and/or anaphase) are capable of undergoing a subsequent mitosis.

## Discussion

**Fig 11:**
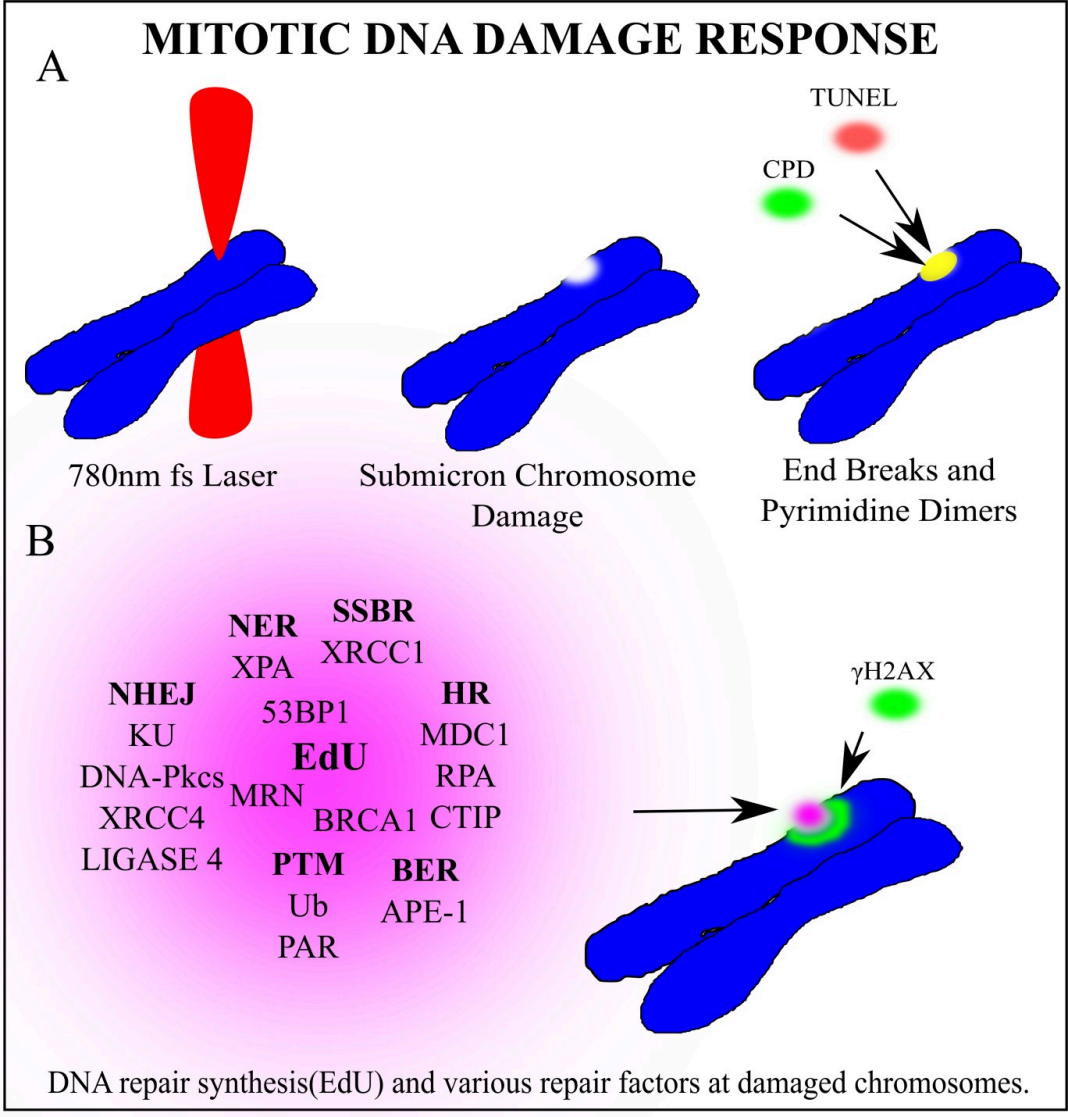
Article summary: Mitotic DNA damage response. (A) A 780nm femtosecond laser was focused to a sub-micron region on a mitotic chromosome. End breaks detected via TUNEL assay and cyclo-butane pyrimidine dimers were found at the laser damage site. (B) Several factors clustered to the damage site. In bold are the repair pathway abbreviations that each factor is most closely associated with. Non-Homologous End Joining (NHEJ), Single Strand Break Repair (SSBR), Base Excision Repair (BER), Homologous Recombination (HR) Post translational modifications (PTM), Nucleotide excision repair (NER). DNA synthesis occurs at the damaged chromosome region as detected via EdU incorporation. Phosphorylated Histone γH2AX on Serine 139 marks double strand breaks and extends from the laser damage spot. Based on the recruitment of the corresponding proteins, we hypothesize that these repair pathways may be activated during mitosis. It should be noted that while factors involved in both early and late steps of NHEJ are readily detectable, only those involved in early step of HR were detected, suggesting that NHEJ may be entirely active while HR may be initiated but not completed during mitosis.

Using the highly focused NIR laser we have determined that the mitotic DNA damage response is more extensive than previously thought. We confirmed that under our conditions the NIR laser induces complex DNA damage, including DNA strand breaks and UV crosslinking damage, Fig 11A. Additionally, we demonstrated DDR-associated post-translational modifications at damage sites, including γH2AX, Ub and PAR, Fig 11B. Furthermore, we detected the recruitment of factors involved in different DNA repair pathways, including DSB and SSB repair, BER and NER, Fig 11B. Together with EdU incorporation at damage sites, our results strongly suggest that these repair pathways are activated. Our evidence also indicates, that while NHEJ may occur, the HR repair process is not completed during mitosis. Our results also suggest mitosis-specific effects of DDR signaling that dictates repair pathway choice, revealing intricate mechanisms of mitotic DNA damage response and repair.

### The laser as a method to elucidate the DDR during mitosis

Previous studies that utilized ionizing radiation and radiomimetic drugs to induce DSBs did not show the accumulation of ubiquitin (Ub), RNF8, RNF168, BRCA1, 53BP1 to mitotic chromosomes [2, 5–14]. However, this might have been due to the limited density of DNA damage, making these studies rely on ionizing radiation induced focus (IRIF) formation of these factors. IRIF are distinct from initial recruitment of the DNA damage-recognition factors and entail further clustering of proteins as well as amplification signals that surround damage sites [50, 68]. Our ability to detect all of the factors is likely due to a higher density of DNA damage in a small submicron volume than these previous studies, thus enabling detection of a high concentration of damage factors. Indeed, Ub and KU have been detected at damage sites on mitotic chromosomes using similar laser systems [28, 38].

### Mitotic DDR may attempt to repair chromosomes by more than one pathway

Our results indicate that HR repair in mitosis is stalled at the Rad51 recruitment step by both CDK1 and DNA-PKcs. This may be due to more than competition between NHEJ and HR. Rather, increased RPA binding and expansion indicate that broken ends are resected, and that chromatin may be prepared for HR repair later in interphase. RAD51 accumulation occurred when either CDK1 or DNA-PKcs where inhibited, indicating that both factors are needed for efficient RAD51 inhibition.

In contrast to stalled HR, factors involved in both early and late steps of NHEJ recruited to damaged chromosomes. Further, KU signal was lower when the DNA ligase signal was greater, strongly suggesting that the NHEJ pathway is activated and likely repairing some of the laser-induced DNA damage. Although cells deficient in DNA-PKcs (a key component of NHEJ) still synthesized DNA, inhibition of PARP (a key player in alt-NHEJ) increased DNA repair synthesis. This suggests that alt-NHEJ is not a major repair pathway in mitosis and that PARP has an inhibitory effect on DNA repair in mitosis, possibly through dictating the repair pathway choice.

Simultaneous inhibition of ATM and DNA-PKcs significantly decreased DNA repair synthesis. This may be due to the ability of both kinases to phosphorylate γH2AX. Therefore, combined inhibition may hinder the recruitment of downstream factors, such as MDC1 [69]. Alternatively, since RAD51 accumulates at damage sites in the absence of DNA-PKcs and ATM is known to mediate HR [61], RAD51-mediated HR repair (other than sister chromatid HR) may contribute to DNA repair synthesis in mitosis. Furthermore, ATM activity was recently shown to be important for NER-mediated secondary DSB repair [70]. Therefore, double inhibition may hinder both primary and secondary DSB repair. While these possibilities are not mutually exclusive, future studies are required to determine whether the entire NER pathway is active in mitosis, and our laser that can induce UV damage would be an excellent tool for this. Taken together, our results suggest that DNA repair synthesis may occur in mitosis by a combination of different repair pathways, which is dictated by DDR signaling and availability of repair proteins.

### Mitotic DNA repair synthesis vs replication stress induced repair synthesis

Previous studies have shown that replication stress-induced DNA damage can lead to DNA repair synthesis in very early prophase but not later phases of mitosis. Cells synchronized in nocodazole did not undergo DNA repair synthesis [16, 17]. However, the mechanism of DNA repair synthesis observed in our study likely differs from those studies in that (1) our damage is inflicted in later prophase, metaphase and anaphase and (2) our damage is not due to active replication stress induced repair. The process described in Bhowmick et al., 2016 and Minocherhomiji et al., 2015 is dependent on Rad52 and MUS81-EME1. Further, Pederson et al. found that under-replicated regions were marked by TopBP1 in mitosis and that ToPBP1 promotes unscheduled DNA synthesis [71]. Therefore, the mechanisms regulating DNA synthesis/repair during mitosis likely depend on the type of damage, quantity and the phase in which damage is induced. It may be interesting to determine whether DNA repair induced in early prophase by means other than replication stress requires some of the same factors mentioned above.

### Damage induced in metaphase is more deleterious for cell division

Under our laser conditions, a significant percentage (25%) of cells damaged in mitosis undergo a second division. Cells damaged in metaphase were less likely to enter a second division in comparison to cells damaged in anaphase or G1. This may be caused by irradiation of a more compacted metaphase chromosome resulting in more DNA damage than in G1. The DNA repair pathway choice in mitosis can be another important factor. A previous study using a different laser (argon ion laser emitting 488 or 514 nm light) showed that cells damaged in mitosis were able to undergo a subsequent mitosis and produce apparently normal cells [26]. This is significant because entry into a second mitosis is indicative of checkpoint recovery and thus DNA repair to the point that it is no longer halting cell cycle progression.

In conclusion, our results show that (1) mitotic cells are capable of DNA repair synthesis, (2) this may be initiated by various repair mechanisms; and (3) is compromised when both ATM and DNA-PKcs are inhibited and is stimulated when PARP is inhibited. (4) Further, a portion of cells damaged in mitosis can undergo sufficient repair to progress into a second division.

## Supporting information

S1 Fig Assessment of the mitotic DNA damage response. (A) The SSB repair factor XRCC1 is found at a laser site which co-localizes with NBS1. A slightly magnified inset of the merged images between XRCC1 and NBS1 is shown on the bottom right. (B)Oxidative base damage in the form of 8- oxo-guanidine was not discernibly higher at laser damaged chromosome regions. On the right is a graph of the pixel intensities of oxidative damage on the laser damage site and outside of the damage site. N = 24 (C) APE-1 was detected on mitotic DNA damage at 5, 10 and 15 minutes post laser. The cell fixed at 15 minutes has two laser damage points. (D) Recruitment of XPA to laser damage created on two different chromosomes within the same cell.
(TIFF)

S2 Fig DNA damage response in different cell lines (M059K, M059J and CFPAC1). (A) Quantification of DNA-PKcs in M059J and M059K demonstrates that the intensity is positive in M059K but not in M059J cells(N = 3). (B) PARylation occurs at damaged chromosome regions. Treatment with 100M NU1025 PARP inhibitor, depicted as PARPi, leads to a decrease in PARylation. Mitotic (N = 5), PARPi Mitotic (N = 3), Interphase (N = 4), PARPi Interphase (N = 4). (C)MO59J cells treated with PARPi are all positive for EdU. (D)A montage depicts a representative cell with γH2AX in green and PAR in red, DAPI in blue. (E) Images of RAD51 accumulation in CFPAC-1 cells during interphase and lack thereof in mitotic cells damaged in mitosis (bottom panel) Scale bar = 10μm. (F)The levels of RAD51 mitotic cells were below or at the same levels of background for CFPAC-1 cells. (G) In a U2OS cell RPA is found on a mitotic cell but not RAD21.
(TIFF)

S3 Fig Box plots of Fig 6A and 6B. (A) Box plots for data in Fig 6A. The same data is presented in these box plots. The range is adjusted in the right one to show the lower points. (B) Box plot of Fig 6B.
(TIFF)

S4 Fig Time to cell division summary and antibody list. (TIFF)

S1 Data Raw values and quantifications (quantifications.xls) that correspond to Figs 1C, 2C, 4A, 5A–5C, 6A, 6B, 7C, 7D, S2A, S2B, S2C and S2F. (XLSX)

S1 Video Movie of cells in 9A. Daughters of a DNA damaged metaphase cell undergo division.
(AVI)

S2 Video Movie of cells in 9B. A DNA damaged metaphase cell undergoes furrow regression.
(AVI)

S3 Video Movie of cells in 9C. Daughters of a DNA damaged anaphase cell undergo division.
(AVI)

S4 Video Movie of cells in 9D. A DNA damaged anaphase cell undergoes furrow regression.
(AVI)

## Abbreviations

DSB: double strand break
DDR: DNA damage response
HR: Homologous Recombination Repair
NHEJ: Non-homologous end joining
NER: Nucleotide Excision Repair
EdU: 5-Ethynyl-2´-deoxyuridine
DNA: PKcsi DNA PKcs Inhibitor
ATMi: ATM inhibitor
PARPi: PARP Inhibitor
