# Supplementary figures and images for: DNA damage induced during mitosis undergoes DNA repair synthesis
Source: PLoS One. 2020 Apr 28;15(4):e0227849. doi: 10.1371/journal.pone.0227849 (PMC7188217; doi:10.1371/journal.pone.0227849)

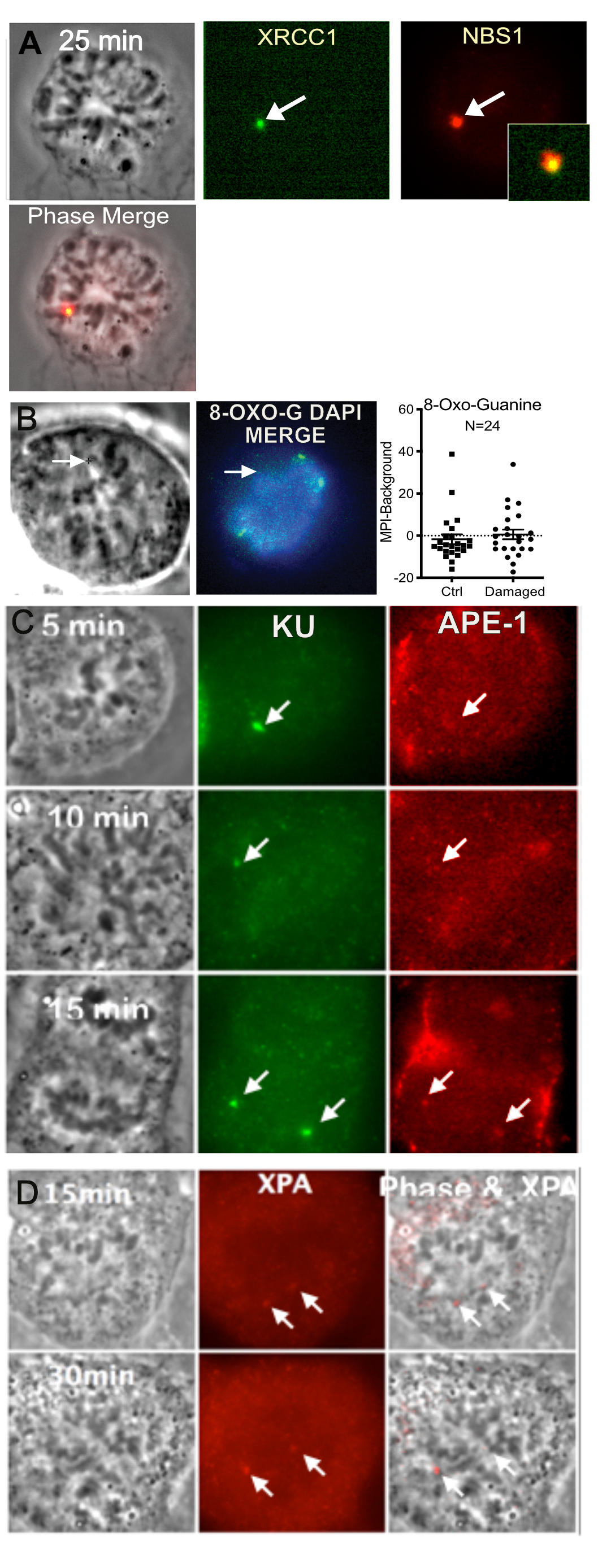

Supplement: S1 Fig — (A) The SSB repair factor XRCC1 is found at a laser site which co-localizes with NBS1. A slightly magnified inset of the merged images between XRCC1 and NBS1 is shown on the bottom right. (B)Oxidative base damage in the form of 8- oxo-guanidine was not discernibly higher at laser damaged chromosome regions. On the right is a graph of the pixel intensities of oxidative damage on the laser damage site and outside of the damage site. N = 24 (C) APE-1 was detected on mitotic DNA damage at 5, 10 and 15 minutes post laser. The cell fixed at 15 minutes has two laser damage points. (D) Recruitment of XPA to laser damage created on two different chromosomes within the same cell. (TIFF) [file pone.0227849.s001.tiff]

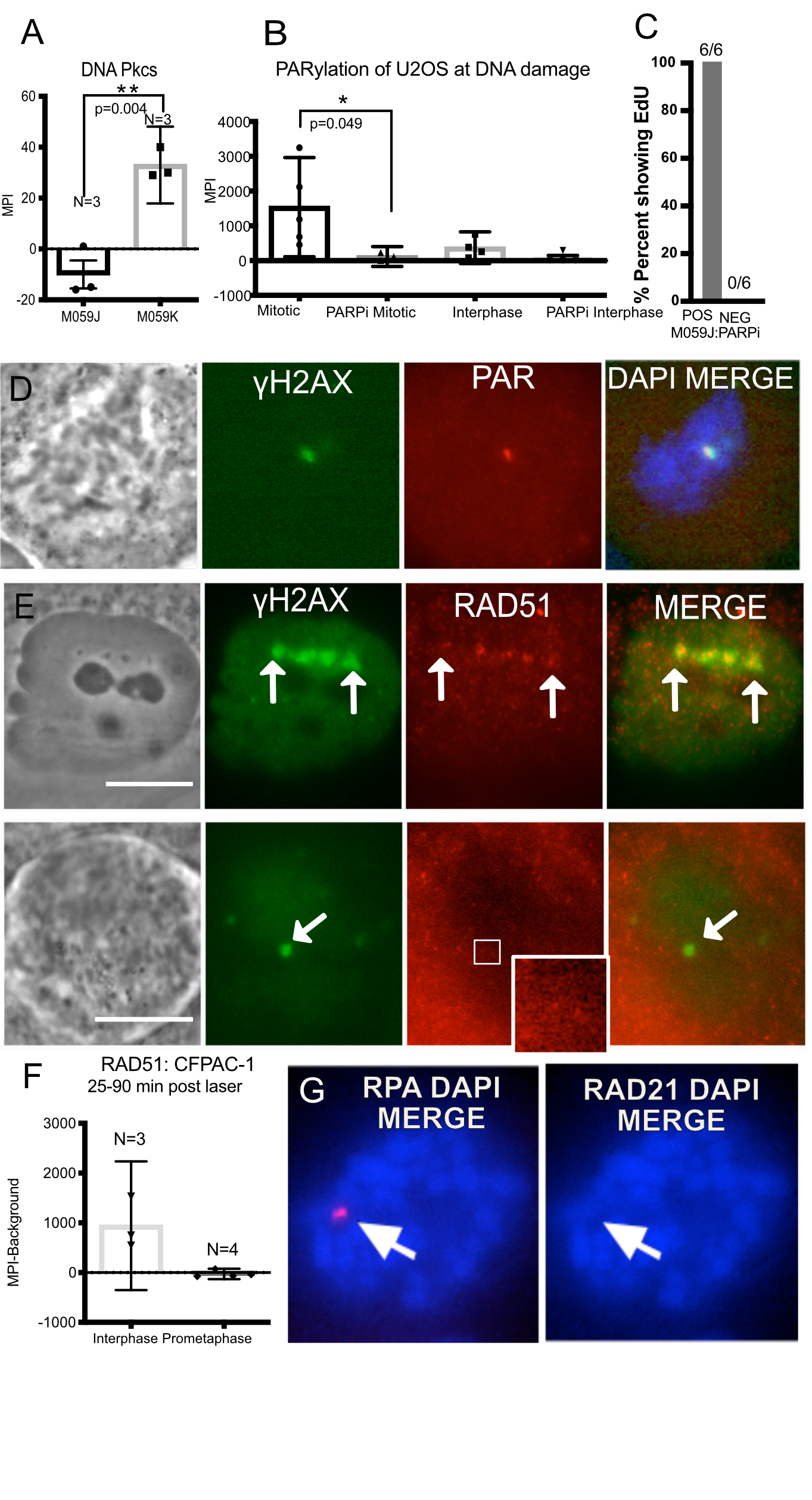

Supplement: S2 Fig — (A) Quantification of DNA-PKcs in M059J and M059K demonstrates that the intensity is positive in M059K but not in M059J cells(N = 3). (B) PARylation occurs at damaged chromosome regions. Treatment with 100M NU1025 PARP inhibitor, depicted as PARPi, leads to a decrease in PARylation. Mitotic (N = 5), PARPi Mitotic (N = 3), Interphase (N = 4), PARPi Interphase (N = 4). (C)MO59J cells treated with PARPi are all positive for EdU. (D)A montage depicts a representative cell with γH2AX in green and PAR in red, DAPI in blue. (E) Images of RAD51 accumulation in CFPAC-1 cells during interphase and lack thereof in mitotic cells damaged in mitosis (bottom panel) Scale bar = 10μm. (F)The levels of RAD51 mitotic cells were below or at the same levels of background for CFPAC-1 cells. (G) In a U2OS cell RPA is found on a mitotic cell but not RAD21. (TIFF) [file pone.0227849.s002.tiff]

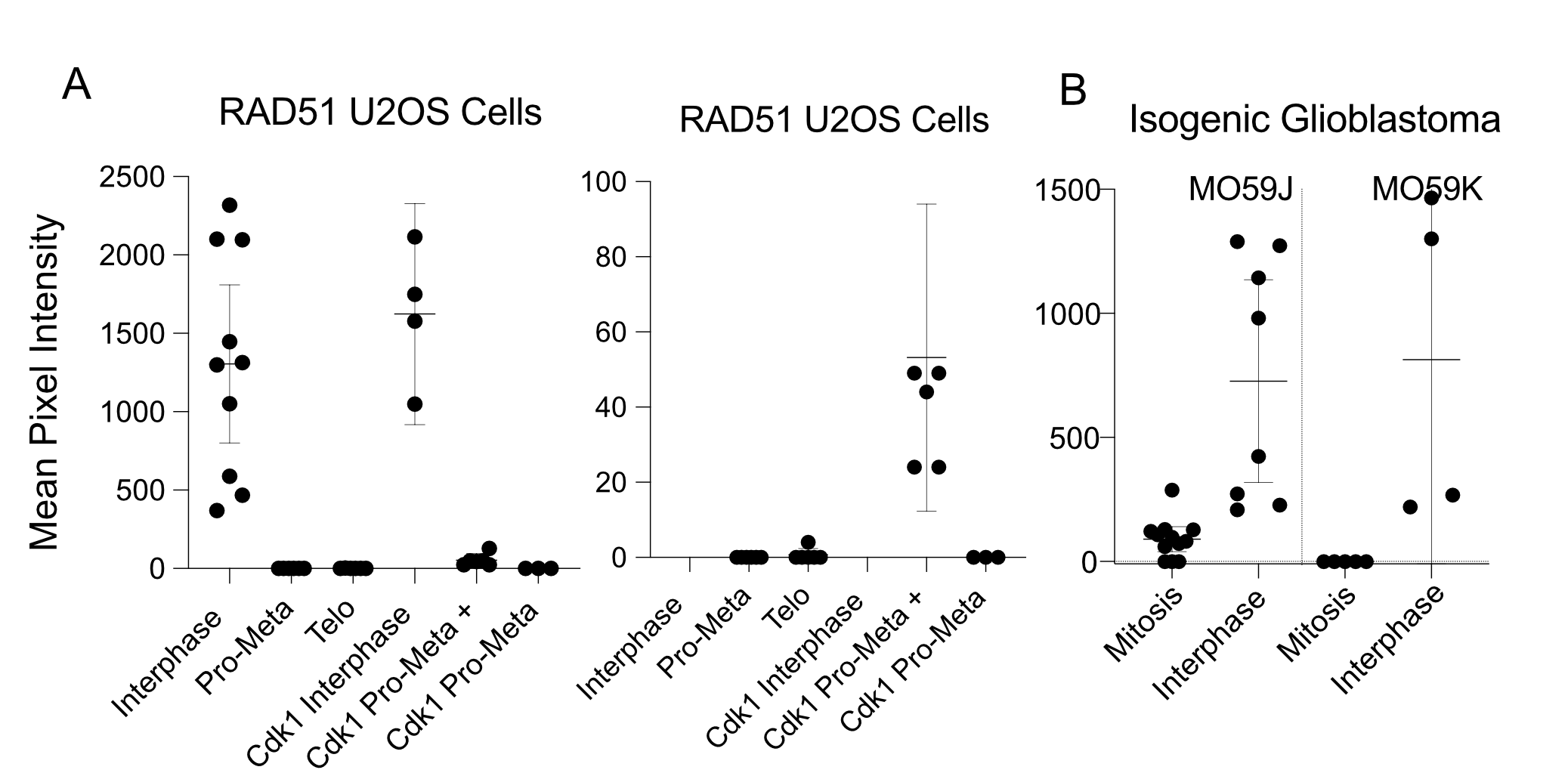

Supplement: S3 Fig — (A) Box plots for data in Fig 6A. The same data is presented in these box plots. The range is adjusted in the right one to show the lower points. (B) Box plot of Fig 6B. (TIFF) [file pone.0227849.s003.tiff]

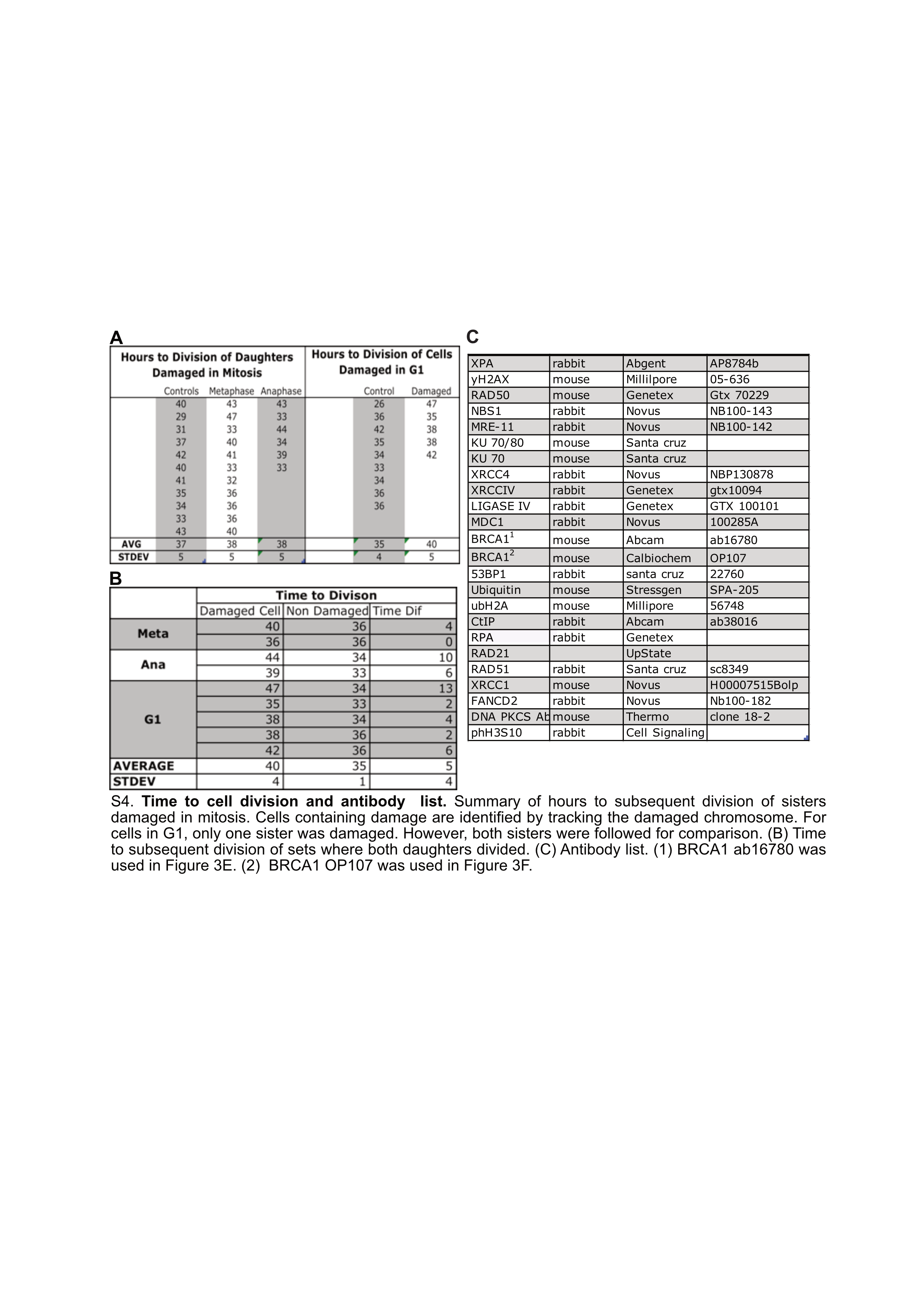

Supplement: S4 Fig — (TIFF) [file pone.0227849.s004.tiff]
